# Five years post whiplash injury: Symptoms and psychological factors in recovered versus non-recovered

**DOI:** 10.1186/1756-0500-3-190

**Published:** 2010-07-13

**Authors:** Daniel Merrick, Britt-Marie Stålnacke

**Affiliations:** 1 Department of Community Medicine and Rehabilitation (Rehabilitation Medicine) Bldg 9A, Umeå University Hospital, Umeå University, SE-901 85 Umeå, Sweden

## Abstract

**Background:**

Few studies have focused on the differences between persons who are recovered after whiplash injury and those who suffer from persistent disability. The primary aim of this study was therefore to examine differences in symptoms, psychological factors and life satisfaction between subjects classified as recovered and those with persistent disability five years after whiplash injury based on the Neck Disability Index (NDI).

**Methods:**

A set of questionnaires was answered by 158 persons (75 men, 83 women) to assess disability (NDI), pain intensity (VAS), whiplash-related symptoms (Rivermead Post-Concussion Symptoms Questionnaire, RPQ), post-traumatic stress (Impact of Event Scale, IES), depression (Beck's depression inventory, BDI) and life satisfaction (LiSat-11).

The participants were divided into three groups based on the results of the NDI: recovered (34.8%), mild disability (37.3%) and moderate/severe disability (27.3%).

**Results:**

The moderate/severe group reported significantly higher VAS, BDI and IES scores and lower level of physical health and psychological health compared to the mild and the recovered groups. Less significant differences were reported between the mild and the recovered groups.

**Conclusions:**

The group with the highest disability score reported most health problems with pain, symptoms, depression, post-traumatic stress and decreased life satisfaction. These findings indicate that classifying these subjects into subgroups based on disability levels makes it possible to optimize the management and treatment after whiplash injury.

## Background

The incidence rate of whiplash injuries in Sweden is estimated to be 1.0-3.2/1000/year [1]. The injuries constitute a major health problem in Western society due to the large number of people with Whiplash associated disorder (WAD) and the high economical costs associated with WAD [2,3] People with acute WAD, mainly complain of neck-pain, stiffness, headache and dizziness. Other symptoms that may occur after the injury are fatigue, concentration and memory problems [2,4]. Most subjects with acute WAD are reported to recover within three months of the trauma [4] however, a significant number of persons experience symptoms several years after the accident [1,5]. Persistent neck-pain has been reported in 84-90% one to two year and in 55% 17 years after the injury [6].

It is still unclear why pain and related symptoms do not resolve after the expected time of healing and which factors are involved in the persistence of symptoms and impairments after the trauma. A bio-psycho-social model is often used to describe the complex interaction of physical and psychological factors in the development of chronic WAD [7]. The long lasting problems after the injury may also interfere with occupational activities, the number of persons on sick-leave or unable to perform their ordinary duties six months after WAD have been reported to vary between 13 and 50% [8,9]. In addition, chronic WAD may also affect leisure and daily life with social contacts and the total experience of life satisfaction [10].

Many studies of long-term problems after WAD have primarily focused on symptoms, especially neck pain in people seeking health care [5,11] but fewer studies have investigated the long-term effects on activity/disability and life satisfaction. In addition, less is known about the differences between subjects who consider themselves as recovered and those who suffer from persistent disability. Sterling et al [12] investigated post-traumatic stress in relation to disability on the NDI during the first six months after whiplash injury. They found that persons who reported themselves to be recovered or to have mild disability six months post trauma reported decreased post-traumatic stress scores in comparison with early after the injury, whereas persons with moderate/severe disability reported persistent post-traumatic stress scores into the chronic stage.

In a scientific as well as in a clinical context, the need of studying subgroups of subjects has been proposed [13,14]. Information about the characteristics of these groups may provide help to develop adequate treatments. Since the levels of disability seems to be of importance in WAD, this study aimed to assess the difference in symptoms, psychological factors and life satisfaction between subjects who were classified as recovered and those who suffered from mild/severe disability based on the NDI [12,15]. In addition, this study examines whether the NDI is a clinically useful tool to classify whiplash disability.

## Methods

A cross-sectional study design was used to study persons five years after whiplash injury. Using the Umeå University hospital's injury and trauma register, data was collected on subjects seeking acute medical assessment for whiplash trauma within three days after the trauma during 2001[16]. Exclusion criteria were fractures/dislocations of the cervical spine related to the injury and seeking medical care after three days. Questionnaires were sent to 304 persons injured during the year 2001 who were between 18 and 64 years old five years after the injury. Most questionnaires were answered by 191 persons; 25 persons actively declined from participating and 158 persons answered the NDI [15] (75 men, 83 women). Demographics, injury characteristics, pain intensity (VAS) [17], whiplash-related symptoms (Rivermead Post-Concussion Symptoms Questionnaire RPQ) [18], depression (Beck Depression Inventory-II) [19], [20] post-traumatic stress (Impact of Event Scale) [21], life satisfaction (LiSat-11) [22], and some additional questions of pain locations and sick leave were related to the results of the NDI [15].

### Statistical analysis

In accordance with the study by Sterling et al [12] the subjects were classified into three groups based on the NDI (recovered ≤ 8, mild disability 10-28, moderate/severe ≥ 30). Data are reported as means with SD, unless indicated otherwise. Kruskal-Wallis and Mann-Whitney tests were used to test differences between groups (i.e. as post hoc tests). X^2 ^test was used to analyse whether groups had different distributions. Multivariate logistic regression was performed to test associations between non-recovered and variables both from the background and five years after the injury (1 = non-recovered; mild and moderate/severe disability; and 0 = recovered). The statistical significant level was set at p < 0.01 (two-tailed).

The study was approved by the ethics committee of Umeå University

## Results

Based on results of the NDI: 34.8% (n = 55) were recovered, 37.3% (n = 59) had mild disability and 27.3% (n = 44) had moderate/severe disability [12,15]. No significant differences were found with respect to gender, age, cause of injury, or information from medical records or the injury and trauma register when comparing those who did not respond to the NDI (n = 33) to the 158 subjects.

### NDI and demographic/injury characteristics

Somewhat higher proportions of women were found in the moderate/severe group (Table 1). The subjects in this group also had the lowest level of education. Only 50% in the moderate/severe group was working or studying five years after the injury and a majority of those on sick-leave related to the injury were found in this group.

**Table 1:** Demographic and injury characteristics

	Recovered	Mild	Moderate/severe	p-value
	n = 55 (%)	n = 59(%)	n = 44 (%)	
Men	28 (50.9)	29(49.0	18(41.0)	
Women	27(49.1)	30(51.0)	26(59.0)	0.581
Age, years, mean (SD)	32.09(10.7)	31.9(12.5)	36.4(11.6)	0.090
Education in years				
9	2(3.7)	7(11.9)	2(4.7)	
10-12	23(42.6)	31(52.5)	27(62.8)	
13-21	29(53.7)	21(35.6)	14(32.6)	0.080
Occupational situation at time of injury				
Working or student	54 (100)	55 (93.2)	39 (90.7)	
Unemployed-seeking work	0	1 (1.7)	1 (2.3)	
Sick-leave full or part time	0	1 (1.7)	4 (9.3)	
Other	0	2 (3.4)	3 (6.7)	0.091
Martial status				
Married, cohabitating	40 (74.1)	40(67.8)	26(60.5)	
Single, divorced or widowed	13 (24.1)	15(25.4)	16(37.2)	
Living with parents	1 (1.9)	4(6.8)	1(2.3)	0.341
Vehicle accidents	43 (78.2)	51(86.4)	31(70.5)	0.139
Position in vehicle:				
Driver	36 (83.7)	38 (74.5)	23 (74.2)	
Passenger front	4 (9.3)	7 (13.7)	6 (19.4)	
Passenger back	2 (4.7)	3 (5.9)	1 (3.2)	
Buss passenger	1 (2.3)	2 (3.9)	0	
Passenger unknown	0	1 (2.0)	1 (3.2)	0.146
Seat belt				
yes	35 (81.4)	39 (76.5)	25 (80.6)	
no	3 (7.0)	5 (9.8)	2 (6.5)	
not applicable	2 (4.6)	6 (11.7)	1 (3.2)	
unknown	3 (7.0)	1 (2.0)	3 (9.7)	0.512
Occupational situation at time of follow-up n = 157				
Working/Student	54 (98.2)	55 (93.2)	22 (50.0)	
Unemployed-seeking work	0	1 (1.7)	1 (2.3)	
Sick-leave full or part time	0	1 (1.7)	21 (47.7)	
Other	0	2 (3.4)	0	< 0.001
Injury reported to insurance company				
yes	21 (38.9)	26(44.1)	30(69.8)	
no	24 (44.4)	16(27.1)	6(14.0)	
don't know	9 (16.7)	17(28.8)	7 (16.3)	0.004

### NDI and pain intensity

The moderate/severe (51.9 ± 26.2 mm) and the mild (19.1 ± 17.4 mm) groups scored significantly higher on the VAS (p < 0.001) than the recovered group (3.6 ± 7.8 mm) and the moderate/severe group scored also significantly higher compared to the mild group (p < 0.001).

### NDI and whiplash-related symptoms

The frequency of occurrence of whiplash-related symptoms was assessed using the RPQ (Table 2). The total score of RPQ differed significantly between the three groups (moderate/severe: 31.9 ± 13.3, mild 15.55 ± 10.9, recovered: 3.78 ± 4.6, p < 0.001).

**Table 2:** Frequency of occurrence of whiplash-related symptoms

Rivermead Post-Concussion Symptoms Questionnaire	Recovered		Mild		Moderate/Severe		
	n = 55	%	n = 59	%	n = 44	%	p-value
Dizziness	10	10.9	22	37.9	35	79.5	<0.001
Nausea/vomiting	3	5.5	14	24.1	23	52.3	<0.001
Sleep disturbance	8	14.5	30	51.7	36	81.8	<0.001
Fatigue	15	27.3	38	65.5	41	93.2	<0.001
Irritability	9	16.4	36	62.1	42	95.5	<0.001
Feeling depressed	9	16.4	24	41.4	35	79.5	<0.001
Feeling frustrated	11	20.0	28	48.3	36	81.8	<0.001
Poor memory	13	23.6	38	65.5	39	88.6	<0.001
Poor concentration	11	20.0	35	60.3	41	93.2	<0.001
Noise sensitivity	5	9.1	28	48.3	31	70.5	<0.001
Blurred vision	6	10.9	20	34.5	27	61.4	<0.001
Sensitivity to light	11	20.0	27	46.6	35	79.5	<0.001
Double vision	1	1.8	8	13.8	16	36.4	<0.001
Restlessness	13	23.6	29	50.0	29	65.9	<0.001
Taking longer to think	10	18.2	27	46.6	39	88.6	<0.001
Headache	10	18.2	31	53.4	40	90.1	<0.001
Total score, mean (SD)	3.8 (4.6)	15.6	10.9)	31.9	(13.3)		<0.001
							
Pain regions							
Neck pain	14	25.9	43	74.1	43	97.7	<0.001
Upper back pain	15	27.8	39	69.6	38	86.4	<0.001
Lower back pain	15	27.8	29	53.7	35	79.5	<0.001

### NDI and depression

The BDI-II was completed by 148 subjects (recovered n = 49, mild n = 57, moderate/severe n = 42, (Table 3)). The recovered and the mild NDI-groups scored significantly lower on the BDI-II (recovered group (2.9 ± 4.4, mild: 6.7 ± 6.3) compared to the moderate/severe: 18.2 ± 9.4; p < 0.001). The BDI-scores of the recovered group compared with the mild group was also significant (p < 0.001). Severe depression level was reported by 11.9% of the moderate/severe group.

**Table 3:** Beck depression inventory (BDI-II)

Level of depression	All subjects	Recovered (R)	Mild (M)	Moderate/Severe (MS)
	(n = 148)	(n = 49)	(n = 57)	(n = 42)
Minimal	110	46	49	15
n (%)	(74.3)	(93.9)	(86.0)	(35.7)
				
Mild	21	3	4	14
n (%)	(14.2)	(6.1)	(7.0)	(33.3)
				
Moderate	12	-	4	8
n (%)	(8.1)		(7.0)	(19.0)
				
Severe	5	-		5
(n%)	(3.4)			(11.9)
				
Total score	8.69	2.86	6.72	18.2
Mean (SD)	(9.18)	(4.35)	(6.29)	(9.39)
				
P-value		R vs M:	M vs MS:	MS vs R:
		< 0.001	< 0.001	< 0.001

### NDI and post-traumatic stress

Table 4 shows differences between the groups on the IES. Post-hoc comparisons showed significant differences between the moderate/severe and the recovered group (p < 0.001) on the total IES score and the subscales Intrusion and Avoidance. When comparing the recovered and the mild groups, there were significant differences on the total IES score (p = 0.019) and the Intrusion subscale (p = 0.008). No difference was found on the Avoidance subscale (p = 0.119).

**Table 4:** Impact of event scale

Level of stress reaction	Recovered (R)	Mild (M)	Moderate/Severe (MS)	P-value
	(n = 54)	(n = 59)	(n = 44)	
Sub clinical (0-8)	42	37	12	
n %	77.8%	67.7%	27.3%	
				
Mild (9-25)	10	13	16	
n %	18.5%	22.0%	36.4%	
				
Moderate (26-43)	2	8	10	
n %	3.7%	13.6%	22.7%	
				
Severe (44-75)	-	1	6	
n %		1.7%	13.6%	
				
Total score	4.91	10.15	21.91	< 0.001
Mean (SD)	(7.06)	(12.39)	(16.87)	
				
Intrusion subscale	2.35	5.03	10.93	< 0.001
Mean (SD)	(3.74)	(6.08)	(8.81)	
				
Avoidance subscale	2.59	5.12	10.98	< 0.001
Mean (SD)	(4.02)	(7.68)	(9.11)	

### NDI and life Satisfaction

The percentage of satisfied on the separate domains [22] are shown in Table 5. (Higher scores on the LiSat-11 indicate higher life satisfaction.) The recovered group scored significantly higher than the mild group on one of the eleven domains; the recovered group scored significantly higher than the moderate/severe group on eight domains. Significant differences were found between the moderate/severe and the mild groups for five of the eleven domains. There were no differences between any of the three groups on the domains contact with friends, family life, partner relationship and sexual life.

**Table 5:** LiSat-11

Life Satisfaction	Recovered (R)	Mild (M)	Moderate/Severe (MS)	R/M/MS	P-value
	Mean (SD)	Mean (SD)	Mean (SD)		
Life as a whole	4.92 (0.93)	4.66 (0.81)	3.75 (1.12)	R vs M:	ns
Satisfied %	71%	59%	23%	R vs MS:	p < 0.001
				M vs MS:	p < 0.001
					
Vocation	4.67 (1.04)	4.20 (1.19)	2.93 (1.65)	R vs M:	ns
Satisfied %	65%	42%	23%	R vs MS:	p < 0.001
				M vs MS:	p < 0.001
					
Economy	4.35 (1.08)	3.86 (1.20)	3.16 (1.54)	R vs M:	ns
Satisfied %	44%	27%	14%	R vs MS:	p < 0.001
				M vs MS:	ns
					
Leisure	4.52 (1.04)	4.07 (1.34)	3.48 (1.49)	R vs M:	ns
Satisfied %	46%	42%	26%	R vs MS:	p < 0.001
				M vs MS:	ns
					
Contacts	4.71 (0.98)	4.53 (1.15)	4.07 (1.21)	R vs M:	ns
Satisfied %	58%	58%	42%	R vs MS:	p < 0.001
				M vs MS:	ns
					
Sexual life	4.47 (1.33)	4.08 (1.63)	3.67 (1.58)	R vs M:	ns
Satisfied %	58%	50%	33%	R vs MS:	ns
				M vs MS:	ns
					
ADL	5.83 (0.47)	5.61 (0.83)	4.68 (1.43)	R vs M:	ns
Satisfied %	96%	93%	61%	R vs MS:	p < 0.001
				M vs MS:	p < 0.001
					
Family life	5.39 (0.62)	5.23 (0.87)	4.92 (1.23)	R vs M:	ns
Satisfied %	94%	81%	70%	R vs MS:	ns
				M vs MS:	ns
					
Partner relationship	5.40 (0.78)	5.21 (1.02)	4.88 (1.34)	R vs M:	ns
Satisfied %	87%	81%	67%	R vs MS:	ns
				M vs MS:	ns
					
Somatic health	4.78 (1.03)	4.02 (1.19)	2.32 (1.16)	R vs M:	p < 0.001
Satisfied %	65%	32%	5%	R vs MS:	p < 0.001
				M vs MS:	p < 0.001
					
Psychological health	5.18 (0.86)	4.75 (0.99)	3.77 (1.25)	R vs M:	ns
Satisfied %	78%	59%	30%	R vs MS:	p < 0.001
				M vs MS:	p < 0.001

### Multivariate regression

The significant variables in a univariate analysis (age, RPQ-symptoms, some LiSat-11 domains, depression, and post-traumatic stress) were analysed in a stepwise multivariate logistic regression model that showed a statistically significant association only between non-recovered and depression (OR = 1.258, CI: 1.166-1.356).

## Discussion

Although previous studies have investigated disability in whiplash patients [23,24], to our knowledge this study is the first to investigate differences in pain intensity, symptoms, post-traumatic stress, depression, and life satisfaction between subjects with persistent disability and subjects classified as recovered in a "non-help-seeking" population long time after whiplash injury.

In the present study the NDI was used to assess and to classify disability in according to a previous study [12]. In their study of whiplash patients six months post trauma, the NDI-scores were slightly lower in comparison with the three subgroups in the present study. Regardless of the time difference between the two studies, it seems possible to assume that the character of disability in persons with WAD around half a year after the injury may persist for longer time. The results on the NDI in our study also agree with the scores reported three years [24] and 17 years [23] after the injury.

In accordance with previous studies of WAD [5], neck pain was the most commonly reported symptom in the moderate/severe and the mild groups. These frequencies were close to results (55%) reported 17 years after the injury [23]. However, pain was also reported in the recovered group, but the frequencies of pain locations (neck, upper and lower back pain) were more equal. Among the whiplash-related symptoms, cognitive deficits with poor concentration and poor memory were unexpectedly high both in the recovered group (25%) and in the moderate/severe and mild groups (60-93%). Since chronic pain [25], depression and post-traumatic stress [26] may affect cognitive symptoms, these factors might have contributed to the cognitive disturbances in all groups in the present study.

The highest post-traumatic stress scores were reported in the moderate/severe group and the frequency of distinct post-traumatic stress reaction (36.3%) was clearly higher than reported in whiplash patients early after injury (13%) [27]. Some evidence for an association between greater post-traumatic stress and late whiplash syndrome [28] has been shown. However, since the levels of post-traumatic stress were high especially in the moderate/severe group, these findings may support the recommendation of early diagnoses and treatment of acute stress to minimize the risk for long-lasting symptoms [29].

Chronic WAD may have a negative impact on quality of life [10]. When comparing life satisfaction on the LiSat-11 between the three disability groups in our study with a large population-based Swedish reference group (2533 subjects) [22], the mild and moderate/severe groups showed lower levels of life satisfaction. However, significant differences were found between the moderate/severe group and the recovered group in eight of eleven domains. Previous research has shown that depression influences outcome for quality of life in chronic WAD [30] and the significantly higher BDI scores in the moderate/severe group may have contributed to their low life satisfaction. Moreover, the association between depression and non-recovered in the multivariate analysis indicates the importance of assessment and treatment of depression in WAD-patients.

Some objections may be raised concerning the findings of this study. Since the variables were investigated in subjects five years after the injury, one cannot rule out that systematic distortions as well as other factors might have influenced the results. Moreover, the subjects in the present study were divided into three disability subgroups based on the NDI. However, as we had no information about the previous levels of disability, there might have been high disability levels in the moderate/severe group before the injury. Although the recovered group also reported presence of symptoms, this might be a result of good coping strategies. Unfortunately, no questionnaires about coping or catastrophic thoughts were included in the study and there was no information about rehabilitation and medication. Fewer people answered the NDI than other questionnaires; a probable reason might be that the NDI was the last questionnaire in a set of several instruments.

## Conclusion

This study has implications for clinicians. Although symptoms often are reported after whiplash injury, the activity levels may differ. Due to the complexity of WAD, the importance of identifying subgroups of WAD has been proposed in order to better tailor their treatment [13,14]. In a previous study, NDI was found to be the most sensitive instrument among several questionnaires to predict poor outcome [24]. Our study adds to previous research: the NDI seems to be a useful instrument for classifying whiplash subjects into subgroups. In general, we found that the group with moderate/severe disability reported high frequency of symptoms, high depression and post-traumatic stress scores and low level of life satisfaction. However, the recovered group also reported symptoms and post-traumatic stress scores, but these levels were not related to disability.

## Competing interests

The authors declare that they have no competing interests.

## Authors' contributions

BMS designed the study. Both authors contributed to the data analyses and to writing of the manuscript.
